# Real‐Time In Vivo Cellular‐Level Imaging During Puncture

**DOI:** 10.1002/advs.202515110

**Published:** 2026-01-05

**Authors:** Huifang Gao, Jiakang Shao, Quanzhi Li, Yizhou Tan, Liangliang Huang, Xiaorong Xu, Ji Qi, Julin Xiao, Wenwen Li, Zhong Wen, Le Wang, Xu Liu, Qing Yang, Ying Gu

**Affiliations:** ^1^ College of Optical and Electronic Technology China Jiliang University Hangzhou China; ^2^ Department of Laser Medicine The First Medical Center Chinese PLA General Hospital Beijing China; ^3^ Laser Medicine Center Hainan Hospital Chinese PLA General Hospital Sanya China; ^4^ State Key Laboratory of Extreme Photonics and Instrumentation College of Optical Science and Engineering Zhejiang University Hangzhou China; ^5^ International Research Center for Advanced Photonics Zhejiang University Hangzhou China; ^6^ ZJU‐Hangzhou Global Scientific and Technological Innovation Center Zhejiang University Hangzhou China; ^7^ Research Center for Frontier Fundamental Studies Zhejiang Lab Hangzhou China

**Keywords:** biopsy guidance, cellular resolution, false‐negative rate reduction, in vivo puncture imaging, microscopic visualization

## Abstract

In the pursuit of precise disease diagnosis, accurate tissue sampling during biopsy is critical. Current CT/ ultrasound‐guided biopsies provide macroscopic localization but lack real‐time cellular‐resolution visualization during puncture, particularly for deep, narrow lumens or small lesions, thereby increasing false‐negative and nondiagnostic sampling risks. It's desired to have a puncture with microscopy imaging. Here, we demonstrate an artificial‐intelligence‐empowered integrative‐light‐field microendoscopy (AIM) needle. This photonic‐mechanically co‐engineered probe overcomes dynamic diffraction limits via a closed‐loop adaptive optics system integrated in a 25G biopsy needle, enabling diffraction‐limited imaging during in vivo puncture. AIM needle resolved characteristic layered microstructures throughout mouse organs (parenchymal/hollow) and pulmonary tumors via synergistic light‐field modulation, leveraging K‐means and convolutional neural networks to enable in situ pathology‐like analysis and tumor/normal tissue discrimination along puncture paths. AIM needle demonstrates dual clinical potential as a complement to macroscopic guidance: potentially providing histology‐like feedback without interrupting procedures while enhancing biopsy targeting accuracy through navigation integration, reducing false‐negative rates in narrow lumens and microlesions, thereby improving early detection sensitivity.

## Graphical abstract

We present an artificial‐intelligence‐empowered integrative‐light‐field microendoscopy (AIM) needle that delivers real‐time in vivo, diffraction‐limited cellular‐level imaging during puncture and visualizes layered microstructures along the needle path. As a microscopic complement to CT/ultrasound, it improves sampling localization and adds preliminary tumor/normal discrimination, potentially reducing false‐negative rates in biopsies of narrow lumens and microlesions, thereby improving early‐detection sensitivity.

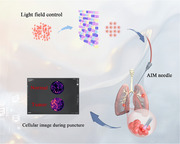

## 1 Introduction

The increasing burden of tumors, characterized by rising incidence and mortality rates, poses a major public health challenge and underscores the urgent need for more accurate diagnostic methods. Puncture biopsy remains indispensable for histological confirmation of malignancy, serving as a cornerstone of current clinical protocols [1]. The accuracy and reliability of biopsy sampling are crucial not only for precise diagnosis but also for devising effective treatment strategies. Clinical puncture guidance imaging technologies, including x‐ray, CT, ultrasound, and magnetic resonance imaging (MRI), play a pivotal role in providing macroscopic localization and guiding biopsy procedures such as percutaneous renal puncture [2, 3, 4, 5, 6, 7], fine‐needle aspiration biopsy (FNAB) of thyroid nodules [8, 9, 10], lung or liver nodule sampling biopsies [11, 12, 13, 14], and cardiac mass‐related interventions [15, 16]. Advanced approaches such as MRI‐guided biopsy of small liver lesions [17], echocardiography‐ or navigation‐guided minimally invasive cardiac interventions [18, 19], and multimodal imaging‐guided techniques further enhance targeting accuracy [19, 20]. Artificial intelligence (AI) algorithms have also made valuable attempts in puncture image localization and trajectory navigation [21, 22]. Despite their widespread use, many clinical imaging modalities provide millimeter‐to centimeter‐scale resolution and are susceptible to physiologic motion (e.g., respiration and cardiac pulsation). These constraints may result in sampling inaccuracies, insufficient tissue acquisition, repeat histopathological examinations, or even secondary surgical interventions, particularly for small or anatomically complex tumors. Each additional sample requires processing extra times, ranging from several hours to days [23, 24]. Such delays escalate healthcare resource burden and heighten risks from treatment postponement and potential tumor progression or dissemination. To mitigate these critical constraints, integrating real‐time microscopic imaging with conventional macroscopic modalities is highly desired in clinical applications. By providing cellular‐resolution data on target lesions, this synergistic approach could achieve enhanced localization precision and help transform diagnostic workflows in interventional oncology.

**FIGURE 1:**
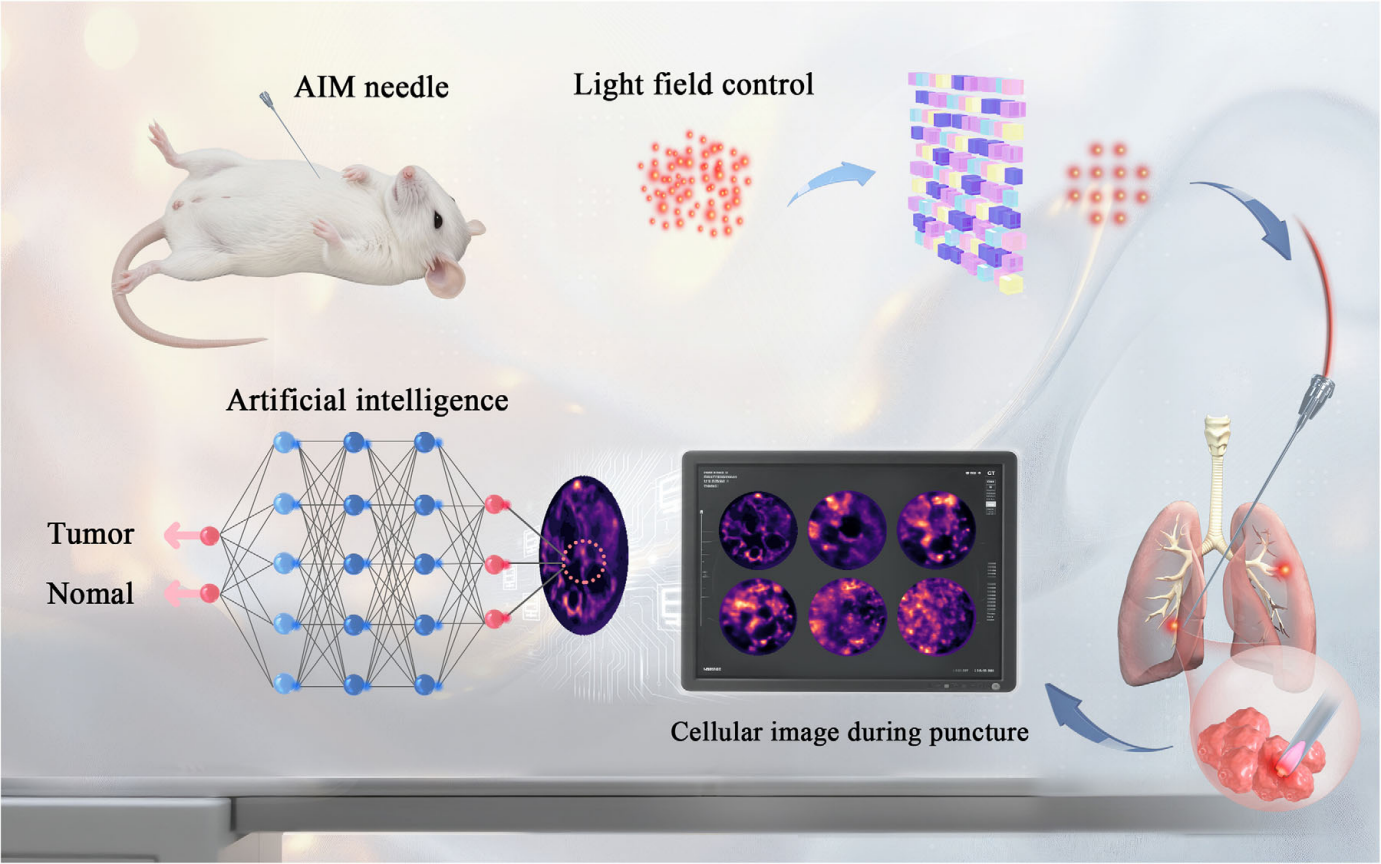
Schematic diagram of the AIM needle. Utilizing light field modulation technology to achieve in vivo microscopic imaging, providing microscopic supplementation to macroscopic guidance (such as CT, ultrasound imaging) for puncture procedures. Leveraging a convolutional neural network (CNN) enables preliminary classification between tumorous and healthy tissues, offering a potential method to reduce false negatives in millimeter‐scale tumor puncture biopsies.

To minimize tissue damage during percutaneous biopsy procedures, the ongoing miniaturization of biopsy needles, as exemplified by the 25‐gauge (25 G) clinical standard featuring an inner diameter of 250 µm, demands imaging probes smaller than 250 µm in diameter, posing significant challenges for concurrent microscopic imaging technology. While emerging modalities such as Stimulated Raman Scattering (SRS) microscopy [25, 26], two‐photon and multiphoton imaging [27, 28], confocal laser endomicroscopy combined with cancer‐targeted near‐infrared (NIR) tracers [29, 30], and short‐wave infrared photothermal microscopy (SWIP) offer exceptional imaging capabilities [31], their clinical translation remains hindered by incompatible probe dimensions. We developed an artificial‐intelligence‐empowered integrative‐light‐field microendoscopy (AIM) needle, overcoming the inherent trade‐off between optical component size and imaging resolution. The AIM probe uses a clinically compatible encapsulation to integrate a 125‐µm multimode fiber (MMF) within a clinical‐standard biopsy needle, establishing an in vivo light‐transmission channel while preserving structural integrity. Through coordinated control of transmission matrix‐based real‐time calibration and focal‐plane optimization, the system achieves diffraction‐limited cellular resolution throughout the puncture trajectory (Figure 1, Supplementary Figure S1). Spatial and frequency‐domain analysis of puncture images enables high‐precision tumor/normal tissue discrimination, while the optimized deep learning architecture accomplishes automated classification. Leveraging its hundred‐micron‐scale footprint and flexible form factor, the AIM needle is poised to integrate seamlessly into existing clinical workflows without altering established procedures, providing cellular‐level imaging capability.

As a single‐use and cost‐effective tool with a silica‐based, biocompatible core, the AIM probe enables real‐time visualization of cellular architecture at puncture sites. During future clinical applications, it assists clinicians in confirming needle‐tip placement within lesion cores—thereby optimizing sampling efficiency and reducing false‐negative rates. By providing complementary microscopic imaging to macroscopic puncture localization, the AIM needle enhances clinical biopsy guidance capabilities. It is expected to minimize tissue trauma and surgical iterations, enhance micro‐lesion detection rate and diagnostic accuracy, and correspondingly reduce complications such as delayed diagnosis and tumor dissemination. In particular, this synergy is poised to establish a dual‐scale macro‐localization and micro‐validation framework that bridges millimeter‐level procedural navigation with micron‐scale tissue characterization, thereby creating a crucial link between macroscopic surgical guidance and microscopic pathological diagnosis without replacing gold‐standard hematoxylin and eosin (H&E) histology. Such an integrated framework lays a technical foundation for advanced biopsy systems.

## 2 Results

### 2.1 Performance of AIM Needle

**FIGURE 2:**
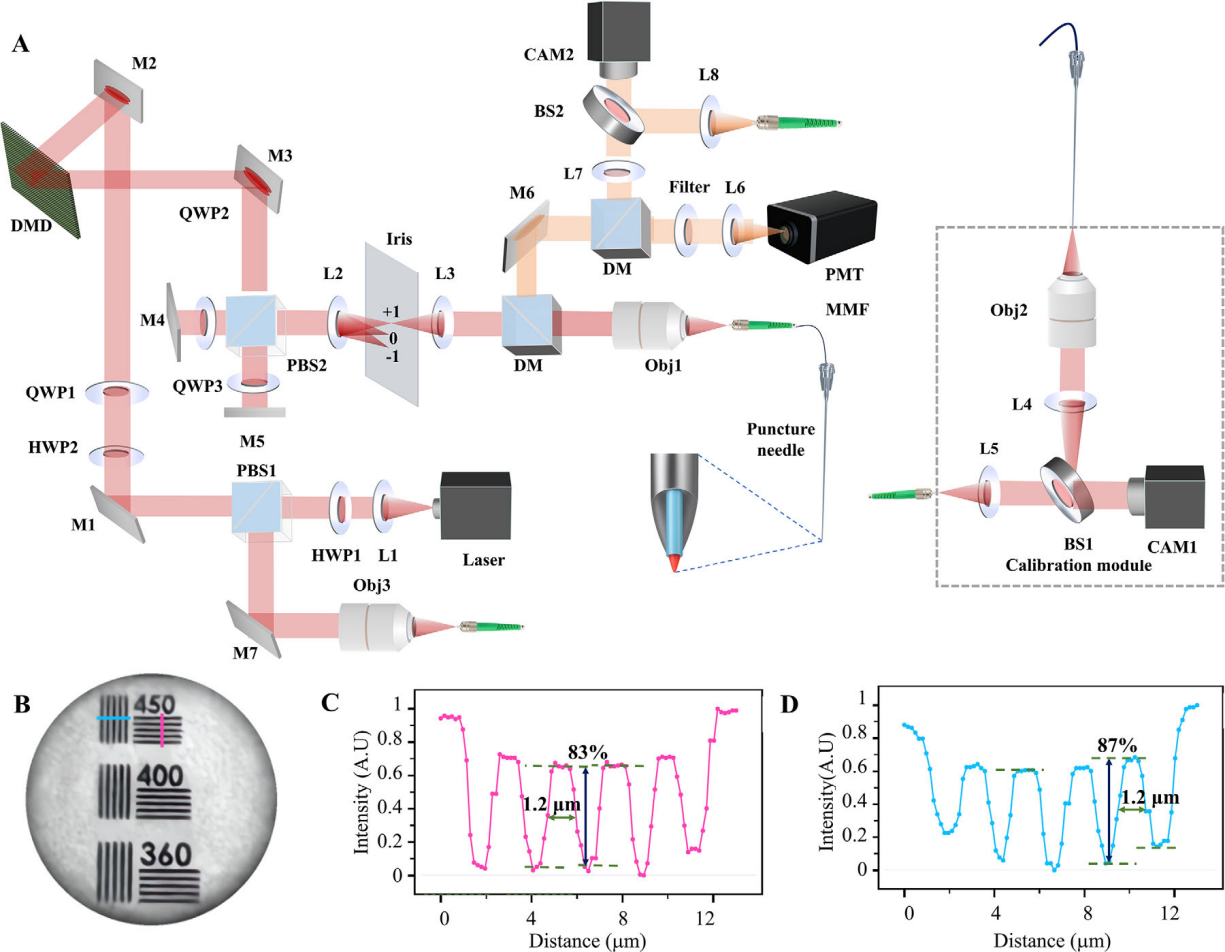
The AIM needle. (A) Schematic diagram of the setup of the AIM needle. M1‐M3: Mirrors; L1‐L6: Lenses; DM: Dichroic Mirror; DMD: Digital Micromirror Device; PBS: Polarizing Beam Splitter; HWP: Half‐Wave Plate; QWP: Quarter‐Wave Plate; OBJ1‐OBJ3: Objective Lenses; PMT: Photomultiplier Tube; CAM: Complementary Metal‐Oxide‐Semiconductor sensor Camera; MMF: multimode fiber; SMF: Single Mode Fiber. (B–D) Resolution characterization of AIM needle. According to the Rayleigh criterion (0.61 λ/NA), the theoretical lateral resolution for the test probe (NA = 0.37) is ≈1.3 µm. (B) Imaging of the resolution test target. (C) Red‐line intensity profiling reveals a minimum resolvable feature size of 1.2 µm (data from Figure 2B). (D) Blue‐line intensity profiling confirms 1.2 µm resolution threshold.

The AIM needle (Figure 2A) utilizes DMD‐generated Lee holographic wavefront shaping and CMOS off‐axis interferometry to dynamically resolve the multimode fiber transmission matrix, achieving dynamic focusing at the fiber facet. The AIM needle establishes a rigid coupling architecture between the MMF probe and needle shaft by integrating a dynamically modulated imaging probe into the inner wall of clinical biopsy needles. The coaxial spatial constraint mechanism reduces relative displacement errors, ensuring precise spatial registration between optical foci and tissue penetration trajectories. Additionally, during puncture procedures, the system retrieves 3D transmission matrices (TM)‐based on current states to image multiple focal planes. By computing image sharpness metrics across planes using the Brenner function, it selects the optimal focal plane to lock the focus, maintaining imaging resolution throughout the puncture process. This design eliminates motion artifacts caused by probe‐needle relative displacement while preserving diffraction‐limited imaging stability, ensuring procedural fluency in percutaneous operations. Repeated puncture experiments showed that the AIM needle maintained stable imaging performance across multiple insertions (Supplementary Figure S2). Furthermore, the hydrophobic coating on the probe's imaging interface effectively mitigates image degradation caused by biological contamination (e.g., blood) during penetration, maintaining imaging clarity through multiple insertion cycles as experimentally verified. Resolution quantification using standardized test targets demonstrated the system's capability of resolving 450‐line pairs per millimeter (lp mm^−1^) with a minimum resolvable feature size of 1.2 µm (Figure 2B–D), where the achieved resolution performance closely approached the theoretical diffraction limit predicted by the 0.37 numerical aperture (NA) probe configuration.

### 2.2 AIM Needle In Vivo Imaging Discriminates Normal and Tumor Tissues

Lung cancer remains the predominant cause of cancer‐related mortality worldwide, creating a critical need for precision early diagnosis of millimeter‐scale pulmonary malignancies through imaging advancements. However, microimaging‐assisted conventional CT‐guided biopsy of millimeter‐scale pulmonary lesions faces persistent challenges, including nonnegligible false‐negative rates attributable to respiratory motion artifacts and sampling inaccuracies, compounded by an inability to resolve microstructural features critical for targeting. The AIM needle may offer a potential approach to this unmet clinical need by providing cellular‐level, real‐time in vivo microscopic imaging to dynamically differentiate heterogeneous tissues (normal versus tumor), thereby offering a promising microscopic‐level complementary pathway. This technology is poised to precision‐constrained clinical guidance modalities by integrating real‐time microvisualization with microimaging, establishing a novel methodological pathway for millimeter‐to‐submillimeter‐scale lesion biopsies, and demonstrating clear potential to reduce false‐negative diagnostic rates. Systematic multiorgan validation encompassing healthy pulmonary parenchyma, pulmonary tumors, solid organs, and gastrointestinal structures confirms the system's capacity to achieve precise microanatomical visualization across varying tissue depths, indicating clinical translational potential as a complementary adjunct to standard biopsy protocols.

**FIGURE 3:**
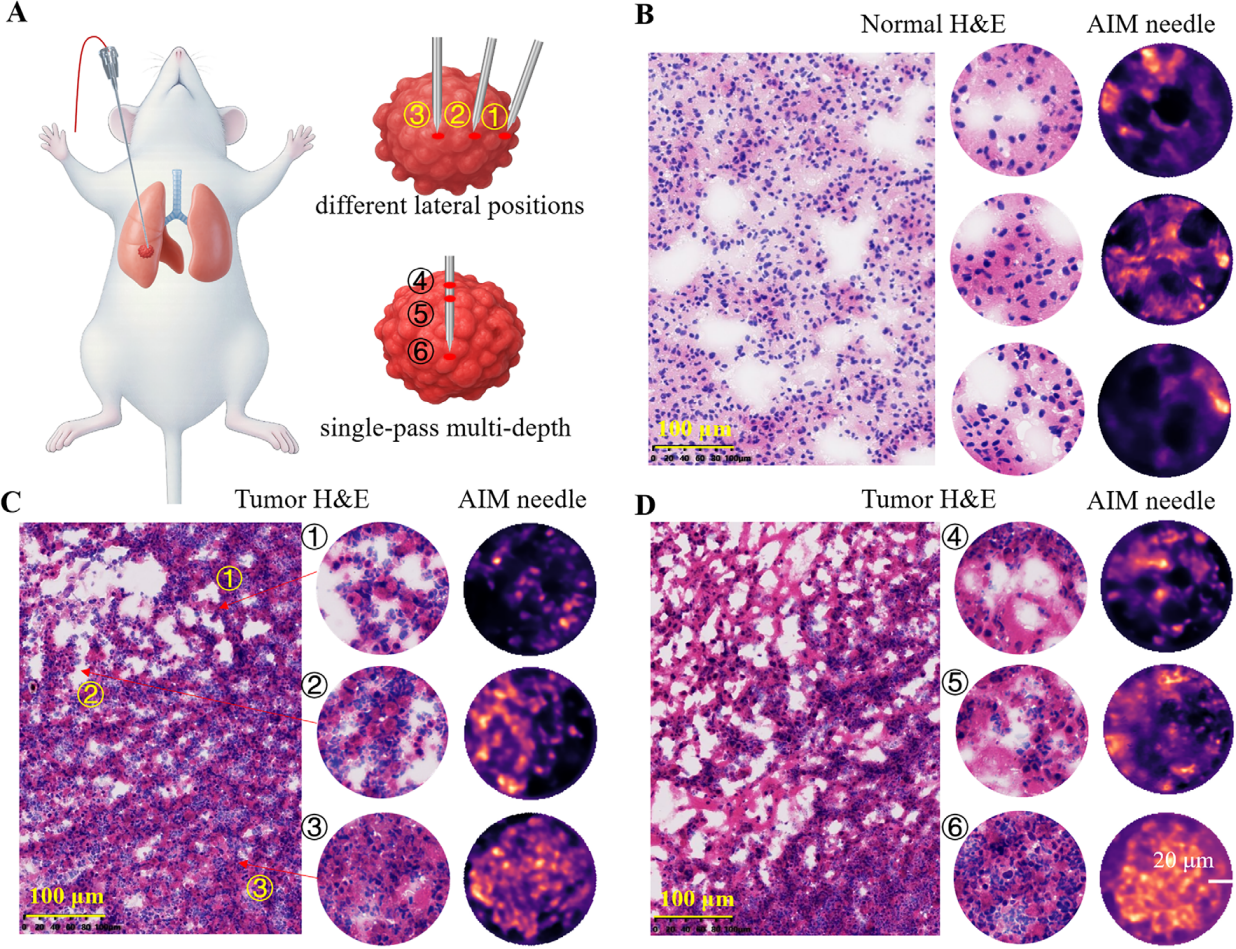
Representative images acquired by AIM needle normal and tumor regions of mouse lungs, along with H&E‐stained sections. The multiple normal‐lung AIM images are from different mice, whereas the lung‐tumor images are from the same mouse. (A) Schematic of in vivo puncture process in mouse lungs. (B) Typical structures of normal lung tissue acquired in vivo by the AIM needle and H&E‐stained histological sections, with regular alveolar structures being visible. (C) Typical structures of different transverse regions of pulmonary tumors acquired in vivo by the AIM needle, as well as H&E‐stained histological sections. (D) Typical structures of pulmonary tumors at different longitudinal depths acquired in vivo by the AIM needle, together with H&E‐stained histological sections. The AIM images reveal changes in tumor structures at different depths longitudinally, with the alveolar structures gradually disappearing from the margin toward the core. Scale bars, 20 µm.

The AIM needle enables real‐time in vivo microscopic imaging with preliminary differentiation capability between tumor and healthy tissues. To demonstrate its performance, in vivo puncture imaging was conducted in normal mice and Lewis lung carcinoma (LLC)‐bearing models, using the clinically approved fluorophore indocyanine green (ICG) to enhance signal‐to‐noise ratio (SNR) and imaging contrast (Figure 3A). AIM needle revealed that when the biopsy needle reached terminal alveoli in normal lung tissue, it encountered regularly shaped, similarly sized round‐to‐oval voids bordered by ICG‐enhanced capillaries (Figure 3B, Supplementary Figure S3A). Gas‐filled alveoli appeared as homogeneous dark regions, while interalveolar septa exhibited marked fluorescence enhancement due to ICG accumulation in perfused capillaries. The inherent alveolar‐interstitial contrast enables real‐time visualization during needle insertion.

As the needle approaches suspicious regions at the tumor margin, alveolar structures become progressively disorganized and irregular. Upon entering the tumor core, the alveolar arrangement is disrupted and may even exhibit a stacked‐like pattern (Figure 3C; Supplementary Figure S3B). With changes in puncture depth, the size and shape of alveoli continue to vary until the alveolar architecture becomes unrecognizable (Figure 3D). During the puncture of tumor tissues at different locations and depths, we documented the progression from disintegrating to obliterated alveolar architectures. AIM needle imaging revealed that tumor cell infiltration leads to progressive effacement of alveolar architectures, rendering alveolar structures indistinct—a marked contrast to normal pulmonary tissue, where intact alveolar structures remain discernible. It should be noted that in actual clinical practice, tumors and normal tissues may lack clear demarcation. Sole reliance on the AIM might fail to achieve ideal differentiation efficacy, thus necessitating integration with multimodal microscopic imaging technologies or other auxiliary modalities to attain accurate discrimination.

### 2.3 Puncture Imaging of Several Murine Internal Organs

**FIGURE 4:**
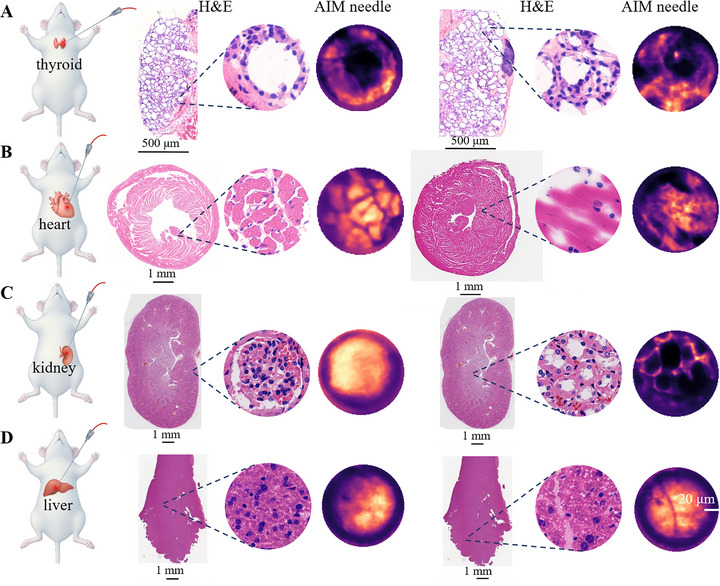
Representative images acquired in real‐time during the puncture process in murine internal organs using the AIM needle, along with H&E‐stained histological sections. This figure includes multiple AIM and H&E image sets; across sets, the sources are as follows: thyroid, different mice; heart, different mice; kidney, same mouse; liver, same mouse. (A) The colloid within the thyroid follicular lumen and the surrounding vacuolar structures acquired in vivo by the AIM needle. (B) The longitudinal and cross‐sectional structures of the cardiac muscle acquired in vivo by the AIM needle were visualized. (C) The AIM needle captured two distinct structures, including the renal glomerulus and the typical thin segments, which are part of the renal tubules. (D) The microvascular channels and lobular structures of the liver acquired in vivo by the AIM needle. Scale bars, 20 µm.

In addition to the lungs, this study extended puncture experiments to multiple murine internal organs, including thyroid, heart, kidney, and liver, to validate the cross‐organ applicability of the AIM needle. During thyroid puncture, the system detected variably sized round/oval structures showing negligible fluorescence, surrounded by fluorescence enhancement zones (Figure 4A). These hypofluorescent areas were hypothesized to correspond to colloid components within thyroid follicular lumens, while the fluorescent peripheral regions corresponded to microvascular‐rich structures. Vascular density gradients during ICG circulation generated differential fluorescence signals between these regions. The spatial distribution characteristics of colloid components and adjacent vacuolar structures captured by the AIM needle showed alignment with H&E‐stained anatomical architectures, supporting its capability to resolve thyroid microstructural features.

The heart, a hollow organ with dense muscular walls, is primarily composed of cardiomyocytes. Penetration of the AIM needle probe enabled clear visualization of myocardial architecture (Figure 4B, Supplementary Figure S3C), revealing two predominant sectional views: longitudinal sections demonstrating parallel alignment of cardiomyocyte bundles, and transverse sections exhibiting polygonal cellular profiles. Subtle regional variations in fiber diameter and spatial density were observed across myocardial regions.

The glomerulus, also known as the vascular ball, comprises capillary tufts within Bowman's capsule. ICG is mainly distributed within blood vessels during circulation, explaining the marked fluorescence enhancement in the central region during puncture imaging (Figure 4C). After the puncture needle penetrates deeper, circular structures can be observed, and these structures are consistent with the thin segments belonging to the renal tubules. As the puncture needle advances further, more loosely arranged structures can be seen, corresponding to collecting ducts (Supplementary Figure S3D).

Upon the penetration of the liver capsule, plate‐like structures with uniform size, a tightly arranged pattern, and strong fluorescence can be observed (Figure 4D). As the needle advances further, intermixed, cord‐like structures become visible. The former are suggestive of hepatic lobules, while the latter are indicative of microvascular channels within hepatic lobules.

### 2.4 Intraluminal Puncture Imaging of the Gastrointestinal Tract

The gastrointestinal wall comprises four distinct histological layers: mucosa, submucosa, muscularis externa, and serosa (with the esophagus having adventitia as its outermost layer), each with specialized physiologic functions. While conventional endoscopy is restricted to visualizing the luminal surface, our AIM needle probe delivered through the endoscopic accessory channel demonstrates the potential for dynamic full‐thickness structural visualization. Within this framework, we implemented depth‐correlated puncture imaging in the murine gastrointestinal tract, capturing layer‐specific architectural features: the mucosa and muscularis exhibited fixed orientations with distinctive textures, whereas the submucosa and adventitia exhibited nonuniform textural organization. These interlayer structural differences support correlating real‐time penetration depth with anatomical stratification during the imaging process.

**FIGURE 5:**
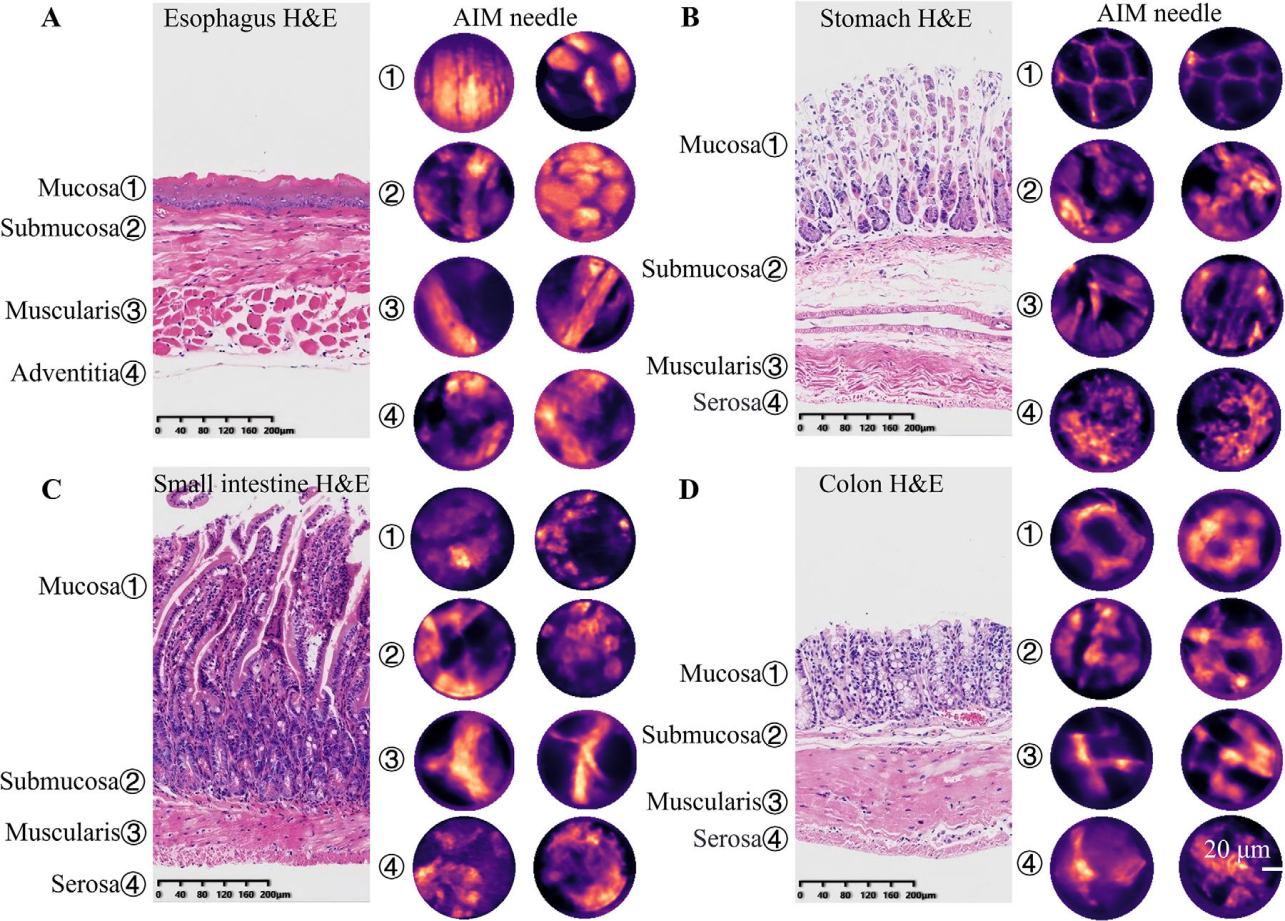
Representative in vivo cross‐sectional images of murine hollow organs acquired from AIM needle are shown alongside longitudinal H&E‐stained sections to illustrate the layered structure. Each panel pairs an H&E reference section with a representative in vivo puncture image from the AIM needle in a layer‐corresponding way, in which the mucosa, submucosa, muscularis, and adventitia are delineated. Gastrointestinal H&E layering is highly consistent across mice, and the H&E sections are shown solely as representative anatomical references for layer identification. (A) Representative structures of the esophagus captured in vivo by the AIM needle include the epithelial structure of the esophageal mucosa, esophageal submucosa, various muscularis layers, and the adventitia. (B) Images of the glandular region of the mouse stomach captured by the AIM needle, showing typical structures of different layers, including the gastric pits in the mucosal layer of the glandular region, as well as deeper lamina propria, muscularis, and adventitia layers. (C) Images of the small intestine highlight the villous structures of the mucosa, the submucosal Brunner's glands, the muscularis layers, and the adventitia. (D) Images of the colon highlight the layered structures of this organ, including the crypt structures of the mucosa, the submucosa, the rich muscularis layers, and the adventitia. Scale bars, 20 µm.

Puncture imaging revealed distinct layer‐specific features in the murine esophagus (Figure 5A). The mucosal layer exhibited band‐like stacked structures attributed to stratified squamous epithelium. Submucosal regions showed transient bright striations corresponding to vasculature within loose connective tissue. At deeper penetration, regularly aligned striations emerged, consistent with muscularis fiber orientation. The adventitial layer displayed vascular signals with structural characteristics mirroring the submucosa.

The mouse stomach consists of glandular and non‐glandular regions, with the glandular stomach further subdivided into the cardia, fundus, and pylorus based on mucosal epithelial cell types. Puncture imaging in the fundus revealed circular, dark, solid structures surrounded by fluorescent signals, suggestive of gastric pits and glands. As the needle advanced, irregular bright signals appeared, indicating the submucosal layer. Further penetration showed strip‐ or fan‐shaped structures, consistent with muscle fibers in the muscularis layer. At the deepest point, bright signals were observed, corresponding to the adventitia, composed mainly of loose connective tissue (Figure 5B).

During puncture imaging of the small intestine, strip‐ and granular‐like fluorescent signal structures were first observed, representing the diverse villus formations in the mucosal layer. As the needle advanced beyond the villi, regularly arranged circular structures appeared in the submucosa, characteristic of Brunner's glands. Further penetration into the intestinal wall revealed strip‐like, orderly arranged, and directional structures, presumed to be the circular and longitudinal muscles of the muscularis layer. Finally, irregular structures were observed, likely corresponding to the loose connective tissue, blood vessels, and other components of the adventitial layer (Figure 5C).

In the process of puncture imaging of the colon, distinct circular, dark structures surrounded by fluorescent signals were initially observed, indicative of colonic crypts. After the puncture needle passed through the crypt structures, strip‐like structures were visible, representing blood vessels and other components in the submucosal layer. As the needle advanced further into the intestinal wall, interwoven, regularly arranged structures appeared, presumed to be the circular and longitudinal muscles of the muscularis layer. Finally, irregular structures were observed, likely corresponding to the loose connective tissue, blood vessels, and other components of the adventitial layer (Figure 5D). These layered structures gradually became visible.

### 2.5 Depth‐Resolved Puncture Imaging of Representative Organs

**FIGURE 6:**
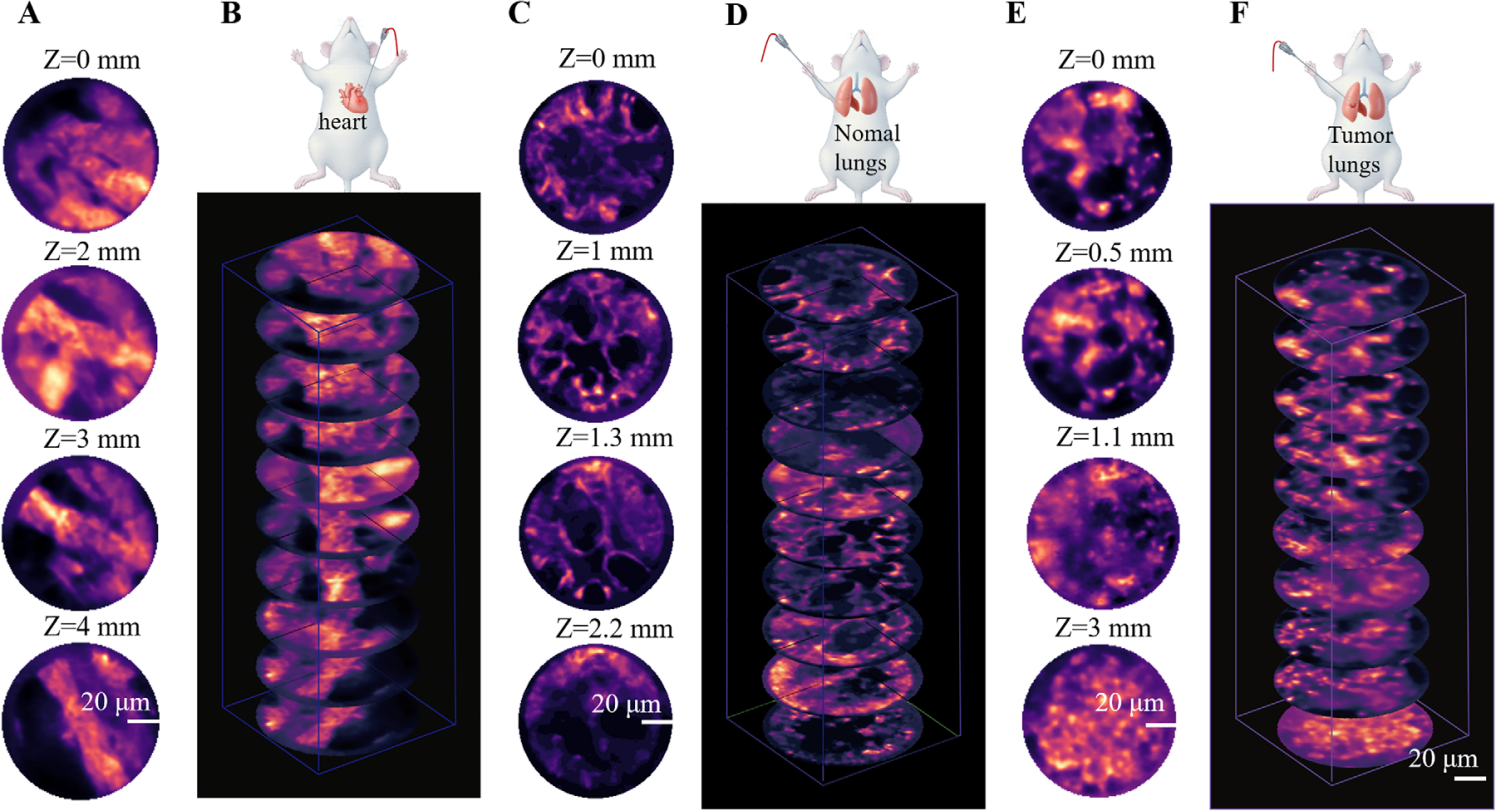
Representative images at different depths of the heart, lung, and lung tumor acquired during puncture. (A) Images of the myocardial structures at different depths are obtained as the puncture needle traverses various positions within the heart, thereby illustrating the characteristics at each depth. (B) Structural characteristics of the heart at different depths are highlighted. (C) Images of normal lung tissue at different depths are presented, which illustrate the characteristics specific to each depth. (D) Structural characteristics of the lung at different depths are emphasized. (E) Progression of the puncture needle through the lung tumor, showing the structural features of the tumor at various depths. (F) Longitudinal schematic diagram of a lung tumor at various depth. Scale bars, 20 µm.

The AIM captured tomographic features at varying penetration depths during tissue traversal. Imaging depths precisely correlated with puncture progression, revealing 3D structural details of cardiac tissue, healthy pulmonary tissue, and pulmonary tumors: myocardial architecture variations during needle advancement (Figure 6A,B); organized microscopic patterns in normal lung parenchyma (Figure 6C,D); and disordered heterogeneity in tumor regions (Figure 6E,F). This depth‐resolved imaging approach provides micrometer‐level spatial guidance for biopsy localization, showing potential to improve precision in diagnostic and therapeutic interventions.

The AIM needle simultaneously acquires real‐time microscopic images at varying depths and records needle advancement during biopsy, thereby establishing a mapping between imaging spatial coordinates and the needle trajectory. The system enables real‐time monitoring of microstructural changes along the puncture path, ensuring high‐precision targeting of lesions. By integrating multidepth and multiposition imaging data, it is expected to reconstruct 3D tumor models that hold potential for predicting tumor infiltration patterns.

### 2.6 Dimensional Disparity between Normal and Tumor Tissues

**FIGURE 7:**
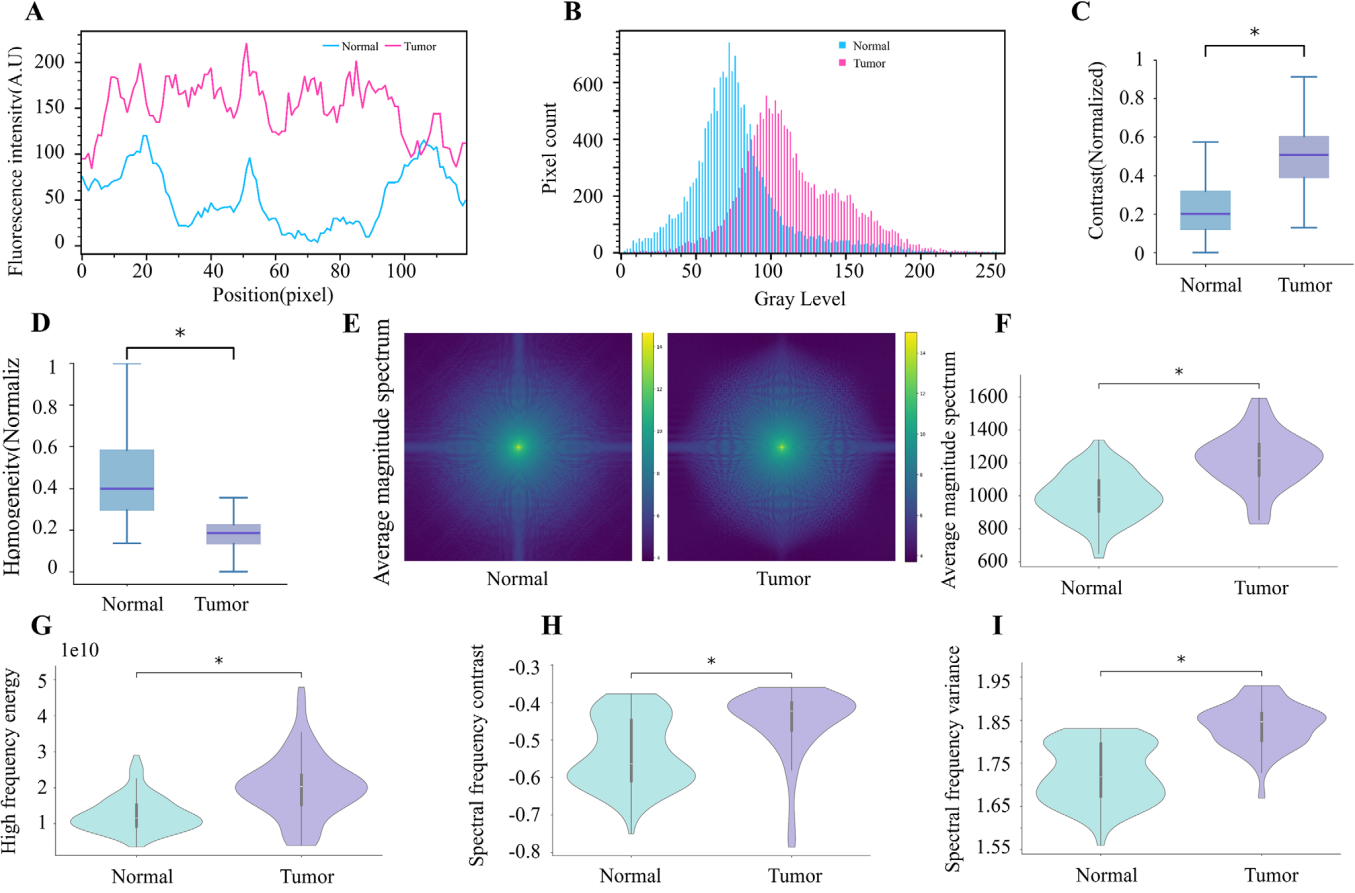
Comparison of optical and spectral characteristics of puncture microscopic imaging between normal lung parenchyma and lung tumors. (A) Comparison of fluorescence intensity between normal lung tissue and tumor regions. (B) Comparison of fluorescence intensity distributions between normal lung tissue and tumor regions. (C) Contrast and (D) Homogeneity analysis of puncture images (min–max normalized; statistical analyses were also performed on the normalized data). (E) Group‐averaged magnitude spectra (AMS) maps of puncture images from normal and tumor tissue. (F) Average magnitude spectrum, (G) High‐frequency energy, (H) Spectral frequency contrast, and (I) Spectral frequency variance. Summary statistics (mean ± SD; *n* = 100 per group) are provided in Supplementary Table S1. Figure annotations indicate Benjamini–Hochberg false discovery rate (FDR)‐adjusted *p* values within the six metrics (*q* < 0.05 threshold): “*” if *q* < 0.05, otherwise “ns” (no graded stars). Primary tests were selected per normality and homoscedasticity (Student's *t*, Welch's *t*, or Mann–Whitney U), and effect sizes are reported as Cliff's delta.

Accurate discrimination between normal lung tissue and tumor images is critical for guiding decisions regarding puncture sampling areas. Fluorescence intensity and grayscale distribution analyses showed that tumor regions had a higher proportion of high grayscale values, whereas normal tissues were mainly concentrated in lower grayscale ranges (Figure 7A,B).

Quantitative analyses of spatial‐ and frequency‐domain metrics further revealed consistent differences between tumor and normal (Figure 7C–I; Supplementary Table S1). In the spatial domain, tumors exhibited significantly higher contrast and markedly lower homogeneity, indicating increased microstructural irregularity. In the frequency domain, the average magnitude spectrum, High‐frequency energy, Spectral frequency contrast, and Spectral frequency variance were all higher in tumors, reflecting greater fine‐scale content and a shifted spectral distribution toward higher radial frequencies. All six metrics met Benjamini–Hochberg FDR control within the figure (*q* < 0.05; stars indicate pass/fail only, no grading), and effect sizes were moderate to very large (Cliff's delta). Collectively, these metrics robustly distinguished tumor from normal tissue, highlighting pronounced spatial heterogeneity and distinctive spectral properties.

**FIGURE 8:**
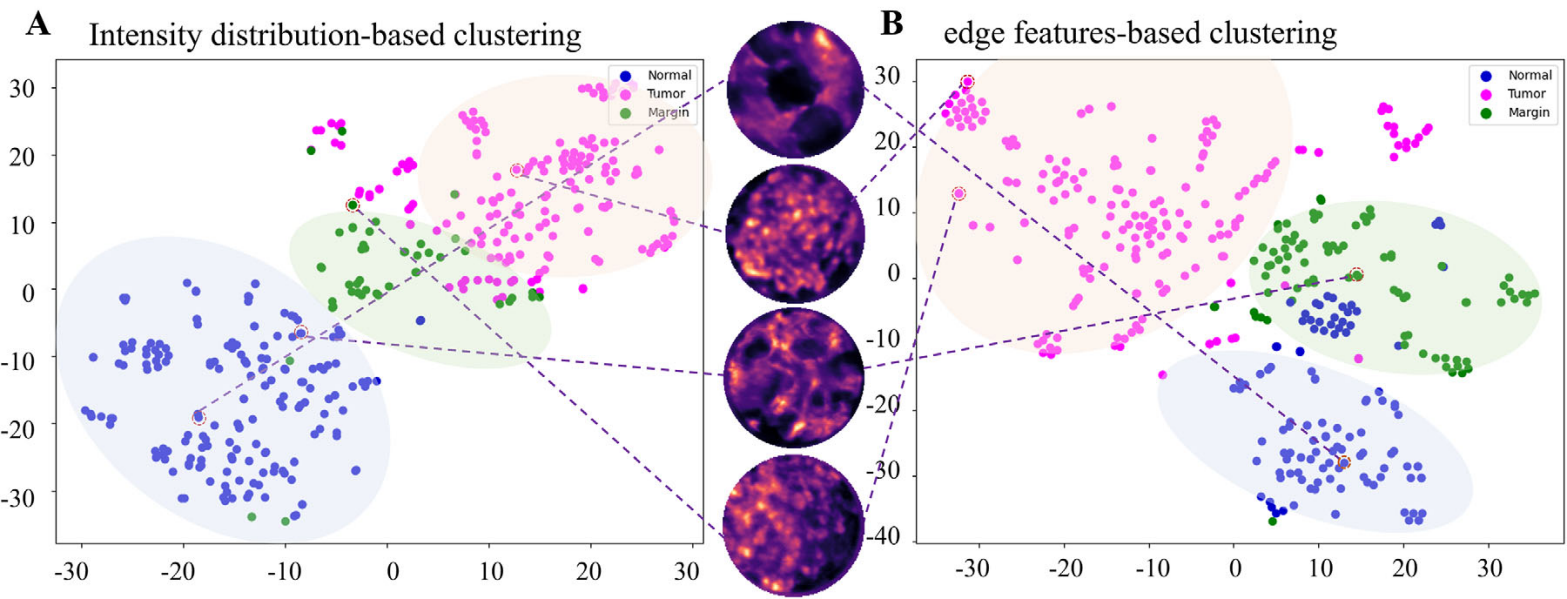
K‐means clustering of tumor and normal tissue images using intensity distribution and edge features (*n* = 387). (A) Intensity distribution‐based clustering. (B) Edge feature‐driven clustering.

This study developed an unsupervised learning framework combining principal component analysis (PCA) for dimensionality reduction and K‐means for clustering, with t‐Distributed Stochastic Neighbor Embedding (t‐SNE) used only for visualization. Utilizing intensity distribution characteristics as the primary dimension, the framework achieves automated classification of puncture images, with visualization results clearly delineating spatial distribution patterns of distinct clusters (Figure 8A). Following feature extraction via multiscale edge detection algorithms, visualization functions were employed to intuitively display clustering outcomes (Figure 8B).

This study employed K‐Means clustering with intensity distribution and edge features, achieving effective differentiation between normal and tumor tissues. However, single‐feature analysis demonstrated diagnostic limitations: intensity distribution‐based clustering achieved high accuracy but misclassified some normal tumor images, while edge feature‐based clustering showed analogous diagnostic discrepancies in tissue interpretation (Supplementary Table S2). Despite this, the two features demonstrated significant complementary effects during classification. Analysis of typical misclassification cases revealed that the vast majority of images misclassified based on structural features could be accurately categorized using intensity distribution characteristics, and vice versa (Figure 8). This synergistic combination of dual‐feature classification proved particularly crucial for reducing false‐negative rates.

**FIGURE 9:**
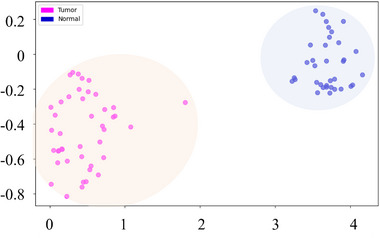
Clustering of tumor and normal images using CNN algorithm (*n* = 387).

To enhance classification accuracy, we leveraged supervised learning mechanisms with CNNs for normal and tumor tissue differentiation. The dataset was evaluated with fivefold stratified cross‐validation, achieving near‐perfect performance. Principal component analysis (PCA) and t‐SNE visualizations verified feature discriminability between healthy/tumor regions, providing reliable support for real‐time puncture judgment (Figure 9).

## 3 Discussion

The AIM needle achieves real‐time in vivo high‐resolution imaging during murine puncture biopsy procedures, enabling cross‐organ visualization. This technology resolves organ‐specific microstructures: alveoli, myocardial architecture, glomerulus, hepatic lobules, and layered structures of hollow organs (e.g., mucosa, submucosa, muscularis, and adventitia). For tumors, it captures margin textural irregularity and core structural disorganization, supporting heterogeneity analysis. Unlike clinical imaging modalities (ultrasound, CT, MRI) that provide only macroscopic puncture guidance [4, 6, 9, 10, 11, 12, 15], AIM needle integrates a 125 µm imaging probe into biopsy needle assemblies, demonstrating potential to achieve dual‐scale visualization (macro‐navigation combined with real‐time cellular‐resolution imaging). This integrated system exhibits potential for precise lesion targeting while acquiring cellular‐level tissue characterization along biopsy trajectories, supplementing a potential new technological pathway for precision interventional diagnostics.

Spatial/frequency domain quantitative analysis of AIM needle imaging revealed distinct differences between tumor and normal tissues (Figure 7). From the spatial perspective, tumor tissue shows higher contrast and lower homogeneity, reflecting intrinsic microstructural disorder—such as structural disruption from abnormal proliferation, disorganized extracellular matrix, and heterogeneous cell density and distribution. These patterns are consistent with malignant progression. In the frequency domain, In the frequency domain, average magnitude spectrum and high‐frequency energy are elevated in tumors, indicating an increase in fine‐scale content arising from complex microstructures and thus stronger high‐frequency components. Spectral frequency variance is higher, indicating broader dispersion of spectral energy across radial frequencies. Spectral frequency contrast (centroid high‐frequency variant) further highlights the shift of energy toward higher radial frequencies. These findings align with tumor heterogeneity studies [32, 33], validating the AIM needle's capability to quantify cellular‐scale pathophysiological features. These quantitative metrics provide objective differentiation criteria that complement traditional visual assessment methods.

In the clustering algorithm, the cascaded architecture integrating unsupervised clustering with a supervised CNN enables step‐change optimization from low‐level feature parsing to high‐level discriminative modeling, supporting real‐time, high‐accuracy classification (Supplementary Figure S4, Supplementary Videos S1 and S2). This technical pathway, synergizing optical characteristics with deep feature extraction, offers a potential mitigation strategy for false‐negative risks in biopsy sampling.

By leveraging the unique properties of MMFs, AIM needle acquires real‐time micron‐scale resolution images at varying depths during biopsy procedures. Through 3D reconstruction, these images generate histology‐like microstructural information, with resolution surpassing that of clinical CT/MRI while supporting intraoperative real‐time acquisition. In future studies, we will adopt strictly uniform axial sampling via motorized, encoder‐synchronized advancement to construct a metrically accurate 3D microlesion map. This 3D microscopic imaging guidance provides detailed spatial relationships of tissue architecture and cellular morphology. Compared to conventional in vivo CT/MRI tomography, the technique significantly enhances spatial resolution; when benchmarked against the gold standard of ex vivo histopathological diagnosis, it achieves real‐time in vivo microscopic scanning diagnostics, thereby breaking through existing technological barriers in diagnostic imaging. Clinically, this method holds potential to assist physicians in direct intraoperative visualization of microstructural features within lesions, improve sampling accuracy for millimeter‐scale lesion biopsies, and provide real‐time preliminary diagnosis through integrated classification algorithms to shorten diagnostic cycles. It should be emphasized that this imaging technology does not serve as a replacement for histopathological examination, but rather aims to provide a noninvasive cellular‐level complementary imaging approach for clinical puncture procedures.

However, the AIM needle has inherent limitations: the forward‐imaging field of view (FOV) is constrained by the scan angle, working distance, and the clear aperture; modestly increasing the working distance can enlarge the FOV; furthermore, although the system currently achieves micron‐level resolution at 785 nm wavelength, optimization of illumination wavelengths and NA could enhance the resolution to submicron levels. Beyond FOV expansion and resolution refinement, a critical direction for future development lies in significantly boosting imaging speed. This can be achieved by implementing high‐speed scanning mechanisms, employing compressed sensing strategies to reduce data acquisition time, and developing feedback‐driven adaptive sampling that prioritizes high resolution for identified regions of interest. These advances collectively aim to shorten operation time and enhance the system's dynamic tracking capability. These refinements would advance clinical translation, providing complementary solutions to macroscale guidance in percutaneous biopsy.

The future development of the AIM needle will follow a logical progression from technical validation to broad clinical application. The initial step will focus on establishing “virtual H&E” studies, enabling a more direct and quantitative correlation between in vivo optical features and pathological benchmarks. Building on this foundation, the integration of tumor‐specific fluorescent probes as key molecular markers is expected to significantly enhance the accuracy of subcellular‐level automated tissue discrimination by improving the specificity of tissue feature recognition. Leveraging these enhanced capabilities, we will then expand the application scope to include multiorgan imaging of varied tumor types (adenocarcinoma, squamous cell carcinoma) across TNM stages I‐IV. This expansion aims to capture stage‐specific pathognomonic imaging signatures, thereby facilitating the construction of a comprehensive oncological image repository. The integration of AIM needle's microscopic imaging with macroscopic biopsy guidance is anticipated to enable dual‐path verification during tissue sampling, thereby systematically reducing false‐negative events through synergistic multiscale interrogation.

## 4 Conclusions

This study developed an AIM needle, overcoming the constraints between minimally invasive intervention and cellular‐resolution imaging through opto‐mechano‐electronic co‐innovation. The probe establishes a stable light‐transmission channel during in vivo puncture and achieves diffraction‐limited resolution throughout the puncture trajectory via real‐time transmission matrix calibration and closed‐loop focal‐plane optimization. Spatial‐frequency co‐characterization combined with deep learning algorithms enables automatic discrimination between tumor and normal tissues. AIM needle holds potential for seamless integration of cellular‐level imaging into standard biopsy workflows, offering a new approach for precise early tumor diagnosis.

## 5 Methods

### 5.1 Experimental Design

This study aims to develop and validate the AIM needle to achieve real‐time in vivo puncture imaging in mice. The experimental subjects include normal mice (*n* = 32) and mice with orthotopic pulmonary tumors (*n* = 6). Using the AIM needle, we perform puncture imaging while recording the puncture depth to establish characteristic structures and layered microstructures at different depths. By comparing with traditional ex vivo cell analysis methods, we evaluate the performance of the AIM needle in real‐time in vivo puncture imaging. The acquired image data will be statistically analyzed and quantified to obtain relevant statistical results. The primary endpoints include successfully obtaining characteristic structures and layered microstructures at different depths, and the secondary endpoint was the discrimination between tumor and normal tissue using quantitative analysis to improve diagnostic efficiency and accuracy.

### 5.2 Performance of the AIM Needle

Both the test probe (NA = 0.37) and the needle‐integrated probe (NA = 0.22, Supplementary Figure S5) provide near‐diffraction‐limited resolution and an approximately hundred‐micrometer‐scale FOV. We prepared an ICG diluent whose emitted fluorescence was detected by PMT under 785 nm excitation. Adjusting the reference‐to‐object light ratio achieved optimal interference effects, thereby maximizing needle imaging contrast. Precise depth control was implemented using a translation stage's fine adjustment knob, followed by fluorescence signal acquisition and image reconstruction via algorithms.

### 5.3 Sample Preparation

This study utilized a total of 38 C57BL/6 mice (4–5 weeks old, approximately 20 g). All mice were maintained under specific pathogen‐free (SPF) conditions. The mice were divided into two groups: 32 healthy mice were purchased from Hangzhou Hangsi Biotechnology Co., Ltd, and 6 tumor‐bearing mice were acquired from Zhejiang Yifen Biotechnology Co., Ltd. The lung tumor‐bearing mice were generated as follows: the tumor cells used were from the LLC cell line (mouse lung carcinoma, Research Resource Identifiers (RRID): CVCL_4358), with cell viability >95% and confirmed to be free of contamination. A 100 µL suspension of tumor cells at a concentration of 1 × 10^7^ cells was injected via the tail vein to allow the cells to reach the lung tissue. Tumors formed within about 3–4 weeks after injection. Of the 6 mice used for modeling, five successfully developed tumors (success rate 83.33%). The tumors were distributed in the lungs, with diameters ranging from 1 to 5.2 mm, indicating inter‐animal variability. In addition, one colon tumor mouse was used (CT26.WT cell line, RRID: CVCL_7256, confirmed to be free of contamination). Among the one mouse modeled, one successfully developed a colon tumor (modeling success rate 100%), with a lesion size of approximately 2.0–4.5 mm at the time of imaging. The final experimental cohort consisted of six tumor‐bearing mice and 32 healthy mice.

### 5.4 Biological Sample Imaging and Postprocessing

To enhance tissue contrast, U.S. Food and Drug Administration (FDA)‐approved ICG with excitation/emission wavelengths of 785/810 nm was used. ICG (final concentration 0.5 mg mL^−1^) was administered via tail vein injection at 0.6 mL for 20 g mice (total 0.3 mg; 15 mg kg^−1^). In vivo fluorescence imaging was conducted within strategically defined organ‐specific time windows based on ICG pharmacokinetic characteristics to ensure consistent signal acquisition (Supplementary Figure S6) [34]. Using the liver—the primary organ for ICG metabolism—as an example, the imaging window is set to 15–30 min after injection. This approach effectively minimizes imaging variations caused by differences in perfusion and excretion rates among different tissues. Pentobarbital sodium ensured immobilization. The probe was mounted inside a 25‐gauge needle, with puncture points and trajectories selected based on target organ depth/location for precise imaging during penetration.

Puncture paths were preplanned to avoid major vessels and facilitate operation; in healthy mice, regions with typical structures were preferentially selected, and in tumor‐bearing mice, the trajectory was directed toward the tumor core. The AIM needle advanced with a controlled pause–observe–advance cadence, and the speed was dynamically adjusted to tissue resistance.

The AIM needle captured real‐time tissue dynamics during needle advancement. Post‐procedure, organs were snap‐frozen at −80°C and transported to the pathology laboratory within 2 h for cryosectioning to validate optical imaging data through H&E staining.

### 5.5 Image Processing and Reconstruction

The image processing pipeline commenced with the calibration of the MMF's transmission matrix. A DMD is utilized to modulate the phase of the incident wavefront of MMF, eliminating or mitigating the optical field distortion introduced by the MMF. The wavefront incident on MMF forms focused spots at different positions on the fiber's exit facet. Through real‐time computation of Brenner sharpness metrics across multiple focal planes, the optimal focal plane is dynamically selected to lock the focus and compensate for cardiorespiratory motion artifacts. These focused spots are then used to perform point‐by‐point scanning of the object. The same MMF collects signal light from the object, which is then received by a high‐sensitivity detector. By utilizing the calibrated transmission matrix to generate addressable focused spots, high‐resolution image reconstruction is achieved through point‐by‐point scanning of the sample and direct mapping of the detected signal intensity at each position to the corresponding pixel value. The reconstructed images were processed by applying a denoising algorithm to enhance the signal‐to‐noise ratio, followed by pseudocolor mapping, and were then cropped to a circular field of view according to the fiber's inherent imaging boundary. Reconstructed images were further processed to generate longitudinal views of needle puncture at different depths, and final illustrations were refined using Adobe Illustrator for presentation.

### 5.6 Statistical Analysis

Spatial features (contrast, homogeneity) were computed from gray‐level co‐occurrence matrices (GLCMs) and min–max normalized across groups. Statistical tests for spatial features were performed on the normalized values. Frequency features from the Fourier magnitude spectrum included average magnitude spectrum, high‐frequency energy, Spectral frequency contrast (log ratio of radial frequencies at 50% and 95% cumulative spectral energy), and spectral frequency variance (log10 of the variance‐to‐mean‐squared ratio of the radial spectral‐energy profile). For each feature, values are summarized as mean ± SD (*n* = 100 per group. Primary tests were chosen per normality (Shapiro–Wilk) and homoscedasticity (Levene): Student's *t*, Welch's *t*, or Mann–Whitney U. All tests were two‐sided, and statistical significance was assessed at a level of 0.05. Multiplicity was controlled within the six metrics using Benjamini–Hochberg FDR (*q* = 0.05); figure stars indicate FDR pass/fail only (“*” if *q* < 0.05, otherwise “ns,” no graded stars). Effect sizes are summarized as Cliff's delta. Analyses were conducted in Python 3.11.

### 5.7 Algorithm

The Real‐ESRGAN denoising algorithm was applied to perform noise reduction on the captured images of resolution test targets [35]. All other AIM needle‐acquired images were processed with the two‐stage deep denoising and edge enhancement framework (TS‐DENet) [36].

This study analyzes image features of normal and tumor tissues using PCA for dimensionality reduction and K‐Means clustering based on intensity distribution and edge characteristics. Images were converted to grayscale, standardized, and reduced with PCA to obtain gray‐level intensity distribution features, while edge features were extracted using Canny and Sobel operators. Both types of features were clustered with K‐means (*k* = 2) in the PCA‐reduced feature space, and t‐SNE was applied solely for 2D visualization.

Additionally, supervised learning clustering is introduced with CNNs, based on the ResNet50 architecture. A tumor and normal tissue classification model was trained and validated using fivefold stratified cross‐validation. The final layer of ResNet50 is modified for the training process, which employs a cross‐entropy loss function and the AdamW optimizer. Model performance was evaluated using accuracy, precision, and recall on the validation and test sets.

### 5.8 Ethics Declarations

All in vivo experiments were approved by the Laboratory Animal Welfare and Ethics Committee of Zhejiang University(NO:30838).

## Conflicts of Interest

The authors declare no conflicts of interest.

## Supporting information




**Supporting File 1**: advs73597‐sup‐0001‐SuppMat.docx.

**Supporting File 2**: advs73597‐sup‐0002‐VideoS1.mp4.

**Supporting File 3**: advs73597‐sup‐0003‐VideoS2.mp4.

## Data Availability

The data that support the findings of this study are available from the corresponding author upon reasonable request.

## References

[1] S. Tummidi , A. Shankaralingappa , and R. Aravindakshan , “Rapid On‐Site Evaluation and Cell Blocks: Getting the Most From the Least Invasive Method in Cytopathology,” Journal of the American Society of Cytopathology 13 (2024): 272–284, 10.1016/j.jasc.2024.04.001. 38702209

[2] Z. Xu , Z. Li , M. Guo , H. Bian , T. Niu , and J. Wang , “Application of Three‐Dimensional Visualization Fused With Ultrasound for Percutaneous Renal Puncture,” Scientific Reports 11 (2021): 8521, 10.1038/s41598-021-87972-8. 33875726 PMC8055667

[3] M. M. Hosseini , A. Yousefi , and M. Rastegari , “Pure Ultrasonography‐Guided Radiation‐Free Percutaneous Nephrolithotomy: Report of 357 Cases,” SpringerPlus 4 (2015): 313. 26155452 10.1186/s40064-015-1078-4 PMC4489965

[4] J. Li , B. Xiao , W. Hu , et al., “Complication and Safety of Ultrasound Guided Percutaneous Nephrolithotomy in 8 025 Cases in China,” Chinese Medical Journal 127 (2014): 4184–4189, 10.3760/cma.j.issn.0366-6999.20141447. 25533819

[5] C. Seitz , M. Desai , A. Häcker , et al., “Incidence, Prevention, and Management of Complications Following Percutaneous Nephrolitholapaxy,” European Urology 61 (2012): 146–158, 10.1016/j.eururo.2011.09.016. 21978422

[6] S. Andonian , C. M. Scoffone , M. K. Louie , et al., “Does Imaging Modality Used for Percutaneous Renal Access Make a Difference? A Matched Case Analysis,” Journal of Endourology 27 (2013): 24–28, 10.1089/end.2012.0347. 22834999

[7] J. J. Rassweiler , M. Müller , M. Fangerau , et al., “iPad‐Assisted Percutaneous Access to the Kidney Using Marker‐Based Navigation: Initial Clinical Experience,” European Urology 61 (2012): 628–631, 10.1016/j.eururo.2011.12.024. 22209052

[8] X. Liu , M. Wang , K. Zhang , H. Zhang , and Y. Lai , “Diagnostic Strategy for Malignant and Benign Thyroid Nodules Smaller Than 10 Mm Based on Surface‐Enhanced Raman Spectroscopy and Machine Learning,” Chemical Engineering Journal 471 (2023): 144794, 10.1016/j.cej.2023.144794.

[9] M. H. Yoo , H. J. Kim , I. H. Choi , et al., “Efficacy of Differential Diagnosis of Thyroid Nodules by Shear Wave Elastography—The Stiffness Map,” Journal of the Endocrine Society 5 (2021): bvab154, 10.1210/jendso/bvab154. 34703960 PMC8533983

[10] M. Radzina , M. Ratniece , D. S. Putrins , L. Saule , and V. Cantisani , “Performance of Contrast‐Enhanced Ultrasound in Thyroid Nodules: Review of Current State and Future Perspectives,” Cancers 13 (2021): 5469, 10.3390/cancers13215469. 34771632 PMC8582579

[11] Q. Wu , B. Cao , Y. Zheng , et al., “Feasibility and Safety of Fine Positioning Needle‐Mediated Breathing Control in CT‐Guided Percutaneous Puncture of Small Lung/Liver Nodules Adjacent to Diaphragm,” Scientific Reports 11 (2021): 3411, 10.1038/s41598-021-83036-z. 33564042 PMC7873283

[12] K. Nakamura , K. Matsumoto , C. Inoue , et al., “Computed Tomography‐Guided Lung Biopsy: A Review of Techniques for Reducing the Incidence of Complications,” Interventional Radiology 6 (2021): 83–92. 35912280 10.22575/interventionalradiology.2021-0012 PMC9327413

[13] Y. R. Huo , M. V. Chan , A.‐R. Habib , I. Lui , and L. Ridley , “Pneumothorax Rates in CT‐Guided Lung Biopsies: A Comprehensive Systematic Review and Meta‐Analysis of Risk Factors,” The British Journal of Radiology 93 (2020): 20190866, 10.1259/bjr.20190866. 31860329 PMC7362905

[14] E. Soylu , K. Ozturk , G. Gokalp , and U. Topal , “Effect of Needle‐Tract Bleeding on Pneumothorax and Chest Tube Placement Following CT Guided Core Needle Lung Biopsy,” Journal of the Belgian Society of Radiology 103 (2019): 21, 10.5334/jbsr.1591. 30972378 PMC6450251

[15] C. Basman , Y. J. Parmar , and I. Kronzon , “Intracardiac Echocardiography for Structural Heart and Electrophysiological Interventions,” Current Cardiology Reports 19 (2017): 102, 10.1007/s11886-017-0902-6. 28879526

[16] S. Tyebally , D. Chen , S. Bhattacharyya , et al., “Cardiac Tumors,” JACC: CardioOncology 2 (2020): 293–311, 10.1016/j.jaccao.2020.05.009. 34396236 PMC8352246

[17] V. F. Schmidt , O. Öcal , V. Walther , et al., “Clinical Benefits of MRI‐Guided Freehand Biopsy of Small Focal Liver Lesions in Comparison to CT Guidance,” European Radiology 34 (2024): 5507–5516, 10.1007/s00330-024-10623-9. 38319427 PMC11364707

[18] A. M. E. Noten , N. M. V. Mieghem , and T. Szili‐Torok , “Remote Magnetic Navigation‐Guided Ventricular Tachycardia Ablation With Continuous‐Flow Mechanical Circulatory Support,” HeartRhythm Case Reports 5 (2019): 217–220. 30997338 10.1016/j.hrcr.2019.01.002 PMC6453544

[19] S. Hu , R. Lu , Y. Zhu , W. Zhu , H. Jiang , and S. Bi , “Application of Medical Image Navigation Technology in Minimally Invasive Puncture Robot,” Sensors 23 (2023): 7196, 10.3390/s23167196. 37631733 PMC10459274

[20] T. Simard , A. El Sabbagh , C. Lane , et al., “Anatomic Approach to Transseptal Puncture for Structural Heart Interventions,” JACC: Cardiovascular Interventions 14 (2021): 1509–1522, 10.1016/j.jcin.2021.04.037. 34294395

[21] X. Hui , P. Rajendran , T. Ling , X. Dai , L. Xing , and M. Pramanik , “Ultrasound‐Guided Needle Tracking With Deep Learning: A Novel Approach With Photoacoustic Ground Truth,” Photoacoustics 34 (2023): 100575, 10.1016/j.pacs.2023.100575. 38174105 PMC10761306

[22] T. Sato , T. Kawai , M. Shimohira , et al., “Robot‐Assisted CT‐Guided Biopsy With an Artificial Intelligence‐Based Needle‐Path Generator: An Experimental Evaluation Using a Phantom Model,” Journal of Vascular and Interventional Radiology 36 (2025): 869–876, 10.1016/j.jvir.2025.01.028. 39848324

[23] K. E. Volmar , M. O. Idowu , R. J. Souers , D. S. Karcher , and R. E. Nakhleh , “Turnaround Time for Large or Complex Specimens in Surgical Pathology: A College of American Pathologists Q‐Probes Study of 56 Institutions,” Archives of Pathology & Laboratory Medicine 139 (2015): 171–177, 10.5858/arpa.2013-0671-CP. 25611100

[24] S. Alshieban and K. Al‐Surimi , “Reducing Turnaround Time of Surgical Pathology Reports in Pathology and Laboratory Medicine Departments,” BMJ Quality Improvement Reports 4 (**2015**): u209223.w3773, 10.1136/bmjquality.u209223.w3773. PMC4693096 26734438

[25] Z. Liu , L. Chen , H. Cheng , et al., “Virtual Formalin‐Fixed and Paraffin‐Embedded Staining of Fresh Brain Tissue via Stimulated Raman CycleGAN Model,” Science Advances 10 (2024): adn3426, 10.1126/sciadv.adn3426. PMC10971418 38536925

[26] J. Ao , X. Shao , Z. Liu , et al., “Stimulated Raman Scattering Microscopy Enables Gleason Scoring of Prostate Core Needle Biopsy by a Convolutional Neural Network,” Cancer Research 83 (2023): 641–651, 10.1158/0008-5472.CAN-22-2146. 36594873 PMC9929517

[27] Z. Qin , Z. She , C. Chen , et al., “Deep Tissue Multi‐Photon Imaging Using Adaptive Optics With Direct Focus Sensing and Shaping,” Nature Biotechnology 40 (2022): 1663–1671, 10.1038/s41587-022-01343-w. 35697805

[28] C. Zhang , et al., “Deep Tissue Super‐Resolution Imaging With Adaptive Optical Two‐Photon Multifocal Structured Illumination Microscopy,” PhotoniX 4 (2023): 38.

[29] G. T. Kennedy , F. S. Azari , E. Bernstein , et al., “Targeted Detection of Cancer at the Cellular Level During Biopsy by Near‐Infrared Confocal Laser Endomicroscopy,” Nature Communications 13 (2022): 2711, 10.1038/s41467-022-30265-z. PMC9114105 35581212

[30] G. T. Kennedy , F. S. Azari , E. Bernstein , et al., “Targeted Detection of Cancer Cells During Biopsy Allows Real‐Time Diagnosis of Pulmonary Nodules,” European Journal of Nuclear Medicine and Molecular Imaging 49 (2022): 4194–4204, 10.1007/s00259-022-05868-9. 35788703 PMC9525441

[31] H. Ni , Y. Yuan , M. Li , et al., “Millimetre‐Deep Micrometre‐Resolution Vibrational Imaging by Shortwave Infrared Photothermal Microscopy,” Nature Photonics 18 (2024): 944–951, 10.1038/s41566-024-01463-6.

[32] J. Shapey , Y. Xie , E. Nabavi , et al., “Optical Properties of Human Brain and Tumour Tissue: An Ex Vivo Study Spanning the Visible Range to Beyond the Second Near‐Infrared Window,” Journal of Biophotonics 15 (2022): 202100072, 10.1002/jbio.202100072. 35048541

[33] F. Yang , Z. Yang , Z. Zhu , et al., “A Joint Photoacoustic Imaging and Broadband Spectral Analysis for Early‐Stage Intraoperative Pathology Assessment: A Case Study With Colorectal Cancer,” Photoacoustics 43 (2025): 100712, 10.1016/j.pacs.2025.100712. 40124587 PMC11929096

[34] G. Zhang , F. Liu , B. Zhang , Y. He , J. Luo , and J. Bai , “Imaging of Pharmacokinetic Rates of Indocyanine Green in Mouse Liver With a Hybrid Fluorescence Molecular Tomography/X‐Ray Computed Tomography System,” Journal of Biomedical Optics 18 (2013): 040505, 10.1117/1.JBO.18.4.040505. 23595825

[35] X. Wang , L. Xie , C. Dong , and Y. Shan , “Real‐ESRGAN: Training Real‐World Blind Super‐Resolution With Pure Synthetic Data,” in 2021 IEEE/CVF International Conference on Computer Vision Workshops (ICCVW) (IEEE, 2021), 10.1109/ICCVW54120.2021.00217.

[36] L. Huang , Z. Wen , Z. Wang , et al., “TS‐DENet: A Transferable Self‐Supervised Learning Method for Multi‐Modal Fluorescence Image Denoising,” Applied Optics 64 (2025): 2534, 10.1364/AO.547303. 40793398

