# Supplementary material for: Real‐Time In Vivo Cellular‐Level Imaging During Puncture
Source: Adv Sci (Weinh). 2026 Jan 5;13(14):e15110. doi: 10.1002/advs.202515110 (PMC12970232; doi:10.1002/advs.202515110)
Supplement: Supplementary file 1 — Supporting File 1: advs73597‐sup‐0001‐SuppMat.docx. [file ADVS-13-e15110-s001.docx]

Supplementary Materials for

Real-time in vivo cellular-level imaging during puncture

Huifang Gao, Jiakang Shao, Quanzhi Li, Yizhou Tan, Liangliang Huang, Xiaorong Xu, Ji Qi, Julin Xiao, Wenwen Li, Zhong Wen*, Le Wang*, Xu Liu*, Qing Yang* and Ying Gu*


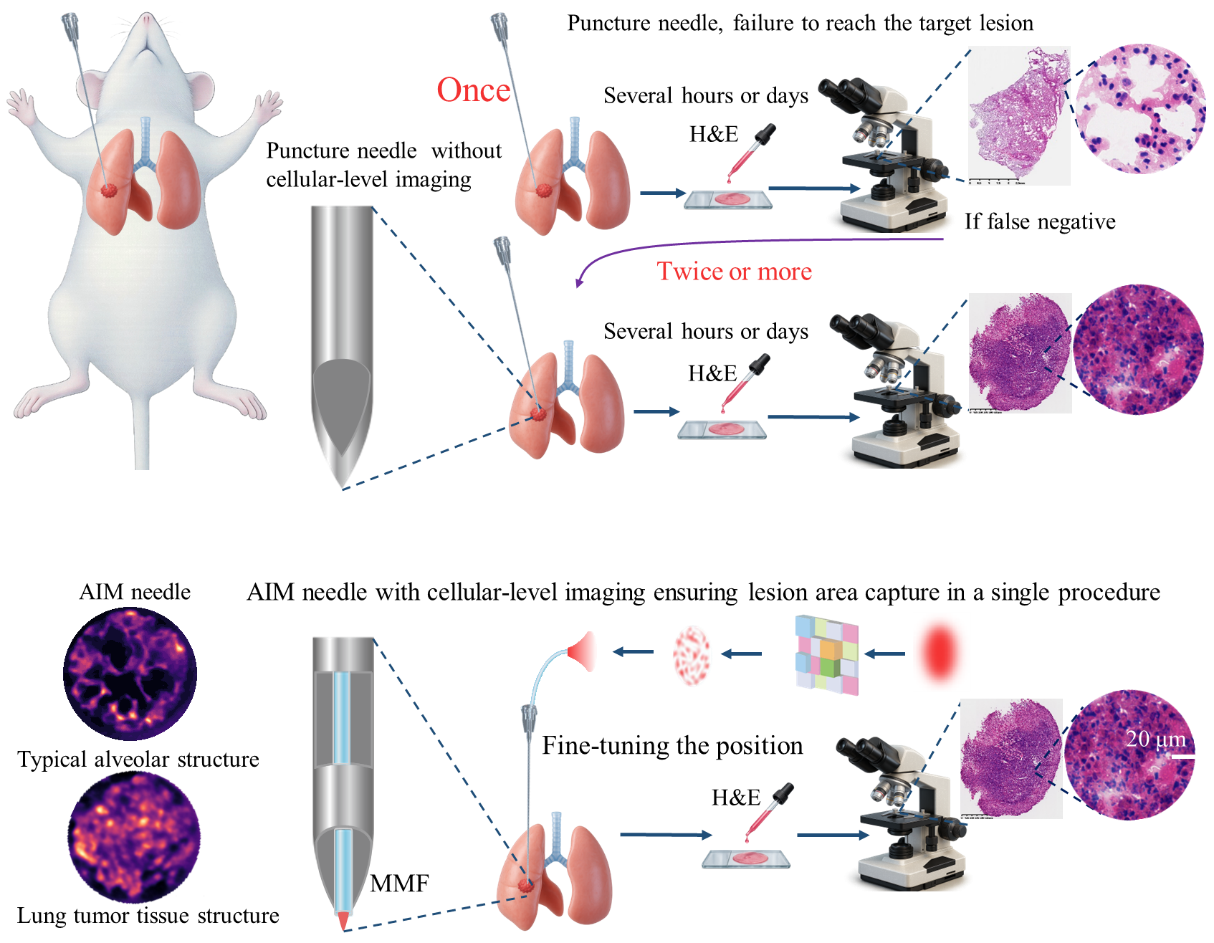


**Supplementary Figure1.** Schematic comparison of puncture biopsies with and without real-time in vivo cellular imaging. Workflow of conventional puncture biopsy: Tissue collection, histological processing (sectioning, staining), histopathological analysis. Sampling inaccuracies may occur during needle insertion, potentially resulting in false-negative diagnoses that may necessitate repeat pathological evaluations or additional surgical interventions. ​Iterative H&E protocols (several hours or days/cycle) cumulatively prolong diagnostic timelines.​

Workflow of puncture biopsy with AIM needle: Achieves microscopic visualization at cellular-level resolution during tissue sampling. Real-time microscopic imaging feedback ensures accurate sampling from target lesions in a single procedure, reducing unnecessary repeat sampling. Scale bars, 20 μm.


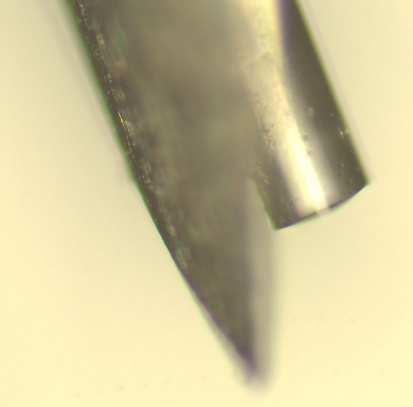

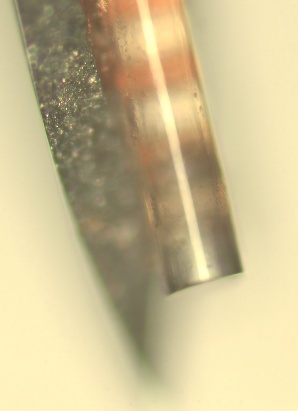


**Supplementary Figure 2.** Microscopic comparison of AIM needle’s probe appearance before and after 50 Insertions


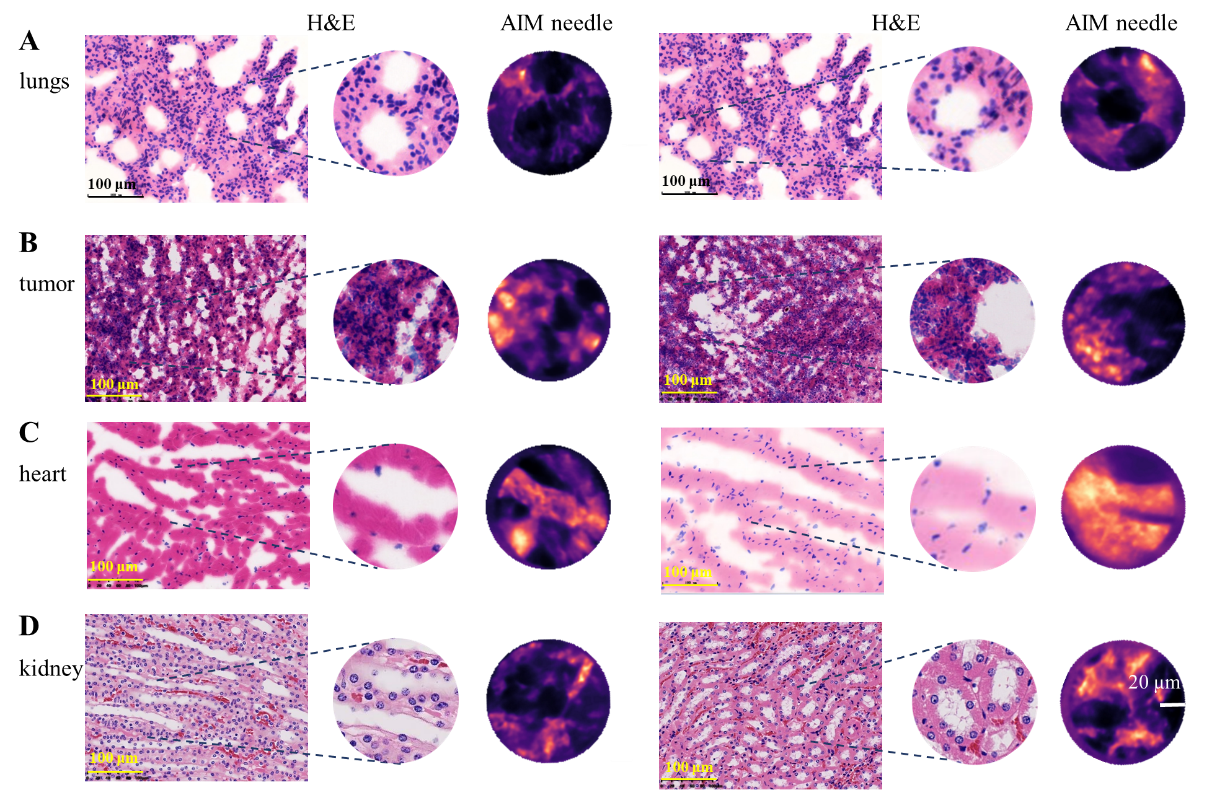


**Supplementary Figure 3.** Images acquired in real-time during the puncture process in parenchymal organs of mice using AIM needle, along with H&E-stained histological sections. This figure includes multiple AIM and H&E image sets; across sets, the sources are as follows: lung, same mouse; tumor, same mouse; heart, different mice; kidney, same mouse. A) Normal alveolar structures within the lungs acquired in vivo by the AIM needle. B) Tumor structures within the lungs acquired in vivo by the AIM needle. C) Longitudinal structures of cardiac muscle acquired in vivo by the AIM needle. D) Partial structures of the renal medulla captured by the AIM needle, including collecting ducts, which are part of the renal tubules. Scale bars, 20 μm.


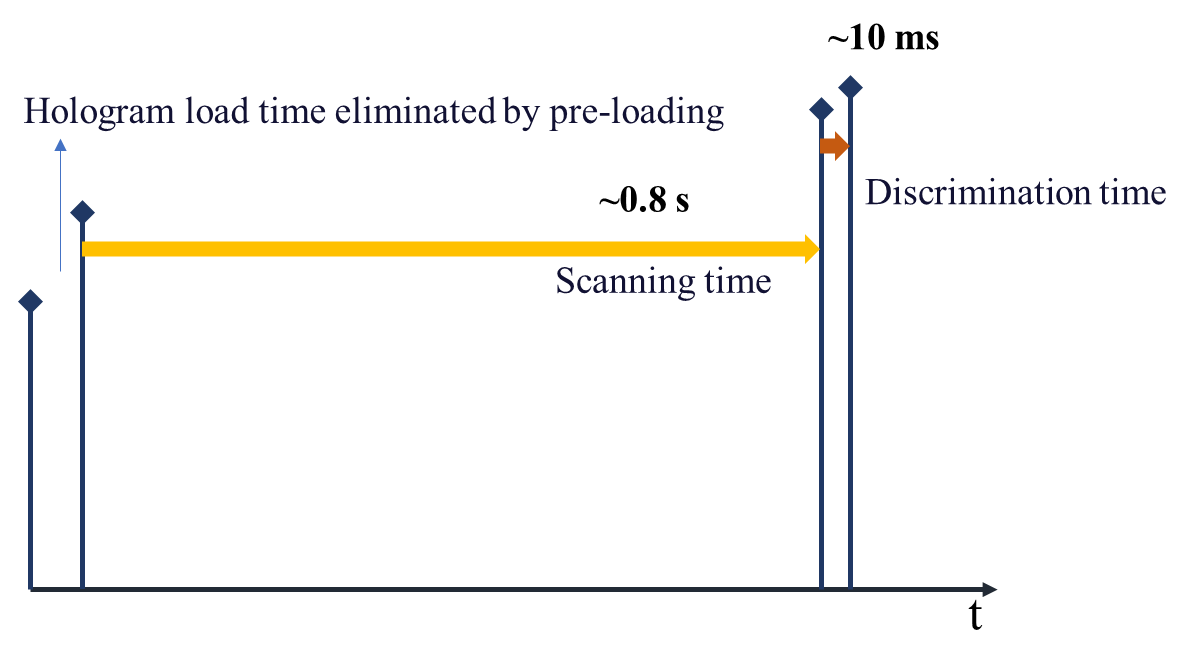


**Supplementary Figure 4.** Schematic diagram of the system timeline


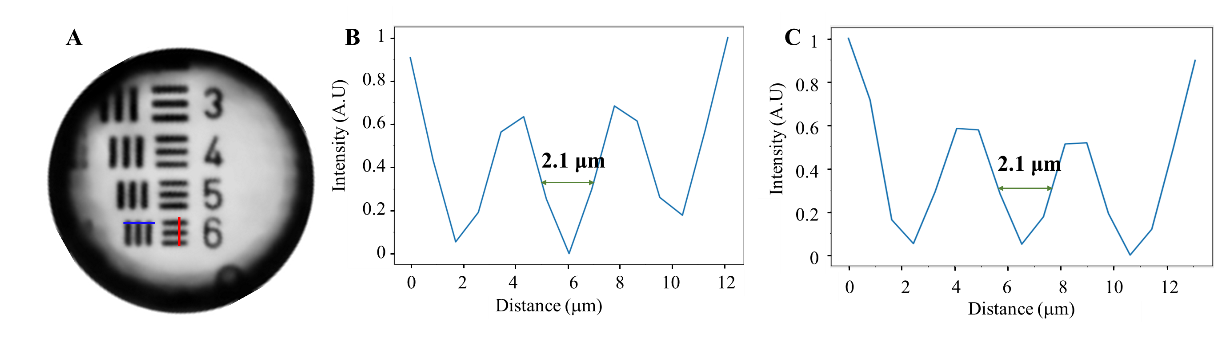


**Supplementary Figure 5.** Resolution characterization with the AIM needle (NA = 0.22). A) Imaging of a resolution test target. B) Red-line intensity profile indicates a minimum resolvable feature size of ≈ 2.1 μm (from panel A). C) Blue-line intensity profile corroborates the ≈ 2.1 μm resolution threshold.


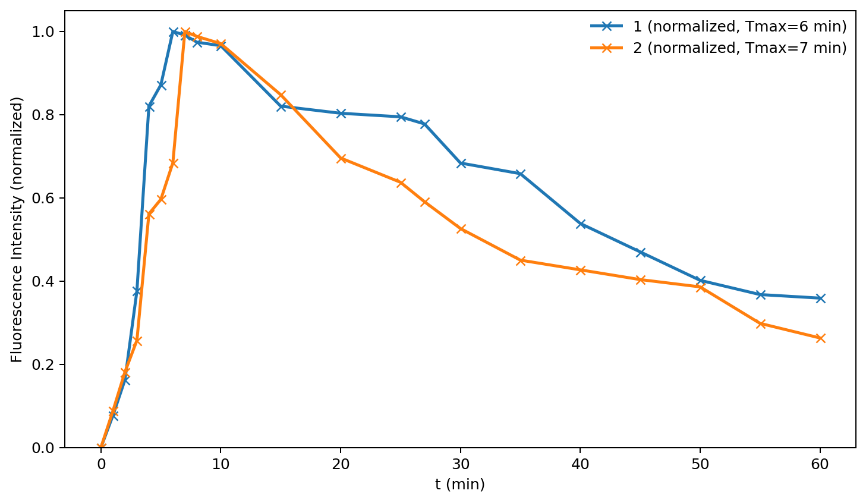


**Supplementary Figure 6.** Time–fluorescence intensity curve of ICG in mouse liver.

**Supplementary Video 1.** Real-time classification applied to existing AIM lung puncture images.

**Supplementary Video 2**. In vivo colon imaging with concurrent real-time classification. Based on training on the colonic mucosal and muscularis layers, the AIM needle distinguishes normal from tumor tissue in real time during imaging. The imaging interface comprises nine windows displayed in chronological order to illustrate the continuous classification process.

**Supplementary Table 1.** Statistical analysis of spatial and frequency domain results

| Feature | Normal (mean ± SD) | Tumor (mean ± SD) | Cliff’s δ |
| --- | --- | --- | --- |
| Contrast (normalized) | 0.24 ± 0.17 | 0.51 ± 0.17 | -0.75 |
| Homogeneity (normalized) | 0.46 ± 0.23 | 0.18 ± 0.08 | 0.85 |
| Average magnitude spectrum | 998.22 ± 142.46 | 1215.92 ± 164.17 | -0.69 |
| High-frequency energy | (1.27 ± 0.49) ×10^10^ | (2.03 ± 0.87) ×10^10^ | -0.58 |
| Spectral frequency contrast | -0.54 ± 0.09 | -0.45 ± 0.09 | -0.53 |
| Spectral frequency variance | 1.73 ± 0.07 | 1.84 ± 0.05 | -0.81 |

**Supplementary Table 2.** Unsupervised K-means clustering compared with pathologists’ accuracy

|  | K-means clustering based on intensity distribution | | K-means clustering based on edge features | | Pathologist 1 | | Pathologist 2 | | Accuracy (%) |
| --- | --- | --- | --- | --- | --- | --- | --- | --- | --- |
|  | Correct | Incorrect | Correct | Incorrect | Correct | Incorrect | Correct | Incorrect |  |
| normal | 171 | 4 | 114 | 61 | 140 | 35 | 154 | 21 | 82.71% |
| tumor | 168 | 44 | 191 | 21 | 173 | 39 | 171 | 41 | 82.90% |
| Total | 339 | 48 | 305 | 82 | 313 | 74 | 325 | 62 | 82.82% |
| Accuracy (%) | 87.60% | | 78.81% | | 80.88% | | 83.98% | | 82.82% |
